# Combined phacoemulsification surgery and intravitreal triamcinolone injection versus stand-alone surgery in patients with type 2 diabetes: a prospective randomized trial

**DOI:** 10.1186/s12886-022-02676-5

**Published:** 2022-11-19

**Authors:** Sarah Zaher Addeen, Iyad Shaddoud

**Affiliations:** Department of ophthalmology, Al Mouassat University Hospital, Damascus University, Damascus, Syria

**Keywords:** Diabetes, Phacoemulsification, Intravitreal, Triamcinolone acetonide, IVTA, Diabetic retinopathy, DR, Central subfield macular thickness, CSMT, Distance corrected visual acuity, CDVA, IOP

## Abstract

**Background:**

We would assess the efficacy of intravitreal injection of triamcinolone acetonide IVTA combined with standard phacoemulsification on the central subfield macular thickness (CSMT), the progression of diabetic retinopathy (DR), and the corrected distant visual acuity (CDVA) in type 2 diabetic patients.

**Methods:**

In this prospective single-blinded randomized clinical trial we recruited patients with type 2 diabetes who were eligible for cataract surgery. The patients were randomly assigned to two groups. The case group received an intravitreal IVTA at the end of phacoemulsification, and the control group had routine surgery. CSMT, progression of DR, CDVA, IOP, and adverse events including endophthalmitis were compared between the groups preoperatively and at 1, 3, and 6 months postoperatively.

**Results:**

Among a total of 66 patients that were treated within the study period, 50 patients were included in the final analysis. The case group comprised 21 eyes, and the control group included 29 eyes. Regression models and corrected ANOVA test for repeated measures showed a significant reduction in CSMT at 3 and 6 months postoperatively, which was most significant when the preoperative CSMT was ≥300 μm, with a cut-off value of 347.3 μm in the case group (*p* < 0.000). DR progression was halted in the case group at 6 months with 52.38% of patients having their DR classified as moderate (*P* = 0.012). CDVA was significantly improved from baseline 6/60 (logMAR 1.0) pre-op to 6/6 (logMAR 0.00) at 6 months post-op in the case group, and from baseline 6/120 (logMAR 1.3) pre-op to 6/12 (logMAR 0.3) at 6 months post-op in the control group. The gain in visual acuity was significantly higher in the case group at all study points (*p* < 0.001). No significant rise in IOP was observed at any study point in both groups (*p* = 0.23 > 0.05). No endophthalmitis was recorded.

**Conclusions:**

Diabetic patients benefit significantly from cataract surgery. This study supports IVTA injection at the end of phacoemulsification in diabetic patients. Triamcinolone is an affordable (which is of particular importance in low-income countries as per our setting), and relatively safe “phaco-enhancer”.

**Trial registration:**

NCT05413330. Initial release 10/06/2022. Unique Protocol ID: UDMS-Opthal-01-2022.

## Background

Cataract and diabetic retinopathy (DR) represent two of the top five leading causes of global impaired vision and blindness according to the WHO [1]*.* Higher incidence and faster cataract progression are well-established in diabetic patients, especially those with higher glycated hemoglobin values [2, 3]. Further, it is estimated that up to 20% of all cataract surgery is performed on diabetic patients [3].

Although the results of cataract surgery in diabetic patients are generally good with modern phacoemulsification techniques, the visual outcomes are suboptimal compared with nondiabetic counterparts due to thicker macula pre and postoperatively [4], and diabetic retinopathy progression [5]. Patients with DR, especially insulin-dependent, seem less likely to achieve a CDVA of 6/6 vision but their visual acuity gain may equal (in sum of lines gained), to those without diabetes [6].

Nevertheless, clinical and laboratory investigations indicate an overall increased level of inflammatory activity [5, 7], and levels of multiple cytokines associated with inflammation and angiogenesis in the aqueous humor in diabetic patients; an inflammatory process that occurs in diabetic eyes as a mechanism of diabetic ophthalmopathy, regardless whether cataract surgery was performed or not [8]. The motive behind using anti-inflammatory and anti-angiogenic factors is that cataract surgery is considered a source of oxidative stress that is not exclusive to diabetic eyes, resulting in macular edema and manipulating normal oxygen levels in the eye in the long term [9, 10]. On the other hand, this assumption may justify the increased incidence of clinically significant pseudophakic cystoid macular edema (Irvine Gass) after uncomplicated cataract surgery from 0.1–2% in the healthy population [11] to 10- 20% [11, 12], or even higher up to 81% in diabetic patients [13]. Moreover, the rates of fluoro-angiographic CME at 1 month and 1 year were 69 and 24% compared with 63 and 0% for nondiabetic eyes, respectively [14]. The differentiation between Irvine Gass syndrome and diabetic macular edema (DME) is not easy. Irvine Gass syndrome represents an inflammatory response, though not fully understood, violation of the blood-aqueous barrier due to surgical trauma and prostaglandins accumulation in the vitreous are accused to be the triggers [15]. On the contrary, DME pathophysiology starts with decreased retinal oxygen tension that manifests as retinal capillary hyperpermeability and increased intravascular pressure mediated by vascular endothelial growth factor (VEGF) upregulation and retinal vascular autoregulation, respectively [16] It was established that DR evolutes in approximately 10–30% of patients after cataract surgery, and that the status of DR at the time of surgery is the most critical predictor for progression [5, 17, 18]. Recently, real-world data have shown enhanced visual outcomes with cataract surgery in diabetic eyes receiving intravitreal therapy (anti-VEGF and corticosteroids) [19, 20].

We aim in this study to investigate the rationale of triamcinolone acetonide injection at the end of phacoemulsification surgery in patients with type 2 diabetes: We hypothesize that IVTA blunts the initiation as well as the progression of diabetic macular edema and diabetic retinopathy, and improves visual outcomes. Besides, we aim to evaluate the consequences and safety of the injection. To our knowledge, this is the first study to investigate the Syrian population in particular.

## Methods

This is a prospective single-blinded (participants only) randomized controlled clinical trial (RCT) with a parallel design. The Institutional Review Board and ethics committee at Damascus University, Damascus, Syria, reviewed and approved the study protocol, and the study adhered to the tenets of the Declaration of Helsinki.

### Patient enrolment

This randomized controlled trial comprised patients 19 years or older, with type 2 diabetes, who had no diabetic retinopathy or mild to moderate non-proliferative diabetic retinopathy at baseline, and were candidates for surgery for visually significant cataracts. No restriction on the preoperative central macular thickness was placed, Computer-generated random numbers were used to randomize patients on a 1:1 ratio. Simple allocation concealment was performed by the closed envelope method: 74 cards had equally either word case/ control written on them. Cards were put in a sealed envelope, and the patient would choose one envelope before surgery. The principal investigator enrolled patients and assigned them to interventions. The second author (IS) who is a vitreoretinal disorders specialist performed the ocular fundus exam. Patients who presented between September 2020 and March 2022 were recruited. All patients provided their written informed consent before inclusion. Only one eye per patient was included in the study and patients were excluded if they were considered functionally monocular as a result of moderate to severe visual impairment in the contralateral eye, as per the definition of the International Statistical Classification of Diseases and Related Health Problems, 10th revision [21].

To avoid selection bias, patients were excluded if they had an increased risk for developing CME in the study eye because of a complication during the current or previous intraocular surgery, intraocular inflammation or uveitis, retinal vein occlusion, or macular pathology that could influence visual function, other than diabetic macular edema. Patients with pseudoexfoliation syndrome, Fuchs endothelial dystrophy, or post-traumatic cataract in the study eye were also excluded. Moreover, patients who presented with severe non-proliferative DR, proliferative DR, or vitreous hemorrhage requiring pan-retinal photocoagulation or vitrectomy, were not considered for participation in the study. Patients who used topical NSAIDs, topical or systemic corticosteroids were excluded, as were patients who received an intravitreal injection with any kind of anti-VEGF in the study eye in the previous 6 weeks: (The effect of bevacizumab, which is the most commonly used in our hospital, appears to wane after 6 weeks as shown in a meta-analysis [22], all anti-VEGFs have half-lives (7 to 10 days) after intravitreal depot injections and clinical durations of action of 4 weeks or slightly more [23]. In addition, anti-VEGFs provided short-term structural protection for 1 month in patients receiving cataract surgery [24]. Intraocular or periocular corticosteroid injection in the previous 3 months was an exclusion criterium, too. Finally, patients were excluded in case of contraindications for any of the investigated drugs, particularly patients with glaucoma, IOP of 21 mmHg or higher, previous steroid-induced IOP elevation, systemic bleeding in the previous 3 months, major systemic surgery in the previous 3 months, or a recent or recurrent cerebrovascular accident, myocardial infarction, or thromboembolic event. Patients who had visually significant preoperative cataract had their fundi evaluated at day one through 1 week postoperatively; according to corneal transparency. It is estimated that macular thickness does not change significantly during this time frame [25]. No Important changes to methods after trial commencement were made.

### Study treatment

All patients had phacoemulsification cataract surgery with implantation of an intraocular lens of the same design in the posterior segment and received perioperative and/or postoperative antibiotics according to the standard of care at Al Mouassat University Hospital. Patients were randomly allocated to treatment groups (IVTA injection/no injection) using computer-generated random numbers. Patients in the triamcinolone acetonide group received an intravitreal injection with 4 mg/0.1 mL preservative-free triamcinolone acetonide that was injected 3.5 mm posterior to the inferotemporal limbus; the injection was given with a 27-gauge needle at the end of cataract surgery. Patients in the control group received no additional treatment at the time of surgery; no sham injections were used in order not to jeopardize the eye to injection complications and risks [26]. The standard post-phacoemulsification treatment protocol in our hospital was tracked in both groups (levofloxacin 0.5 mg\ ml eyedrop q.2.h for a week, followed by four-time daily over a week plus prednisolone acetate 1% q.2.h for a week, followed by a weekly tapering over 3 weeks as follows: 6-time, 4-time, twice daily, respectively). Patients were not informed about the applied treatment until 4 weeks postoperatively.

### Outcome assessments

Changes in CSMT and DR progression were designated as the primary outcomes and as measurements of efficacy. Other investigated outcomes were CDVA (as measurements of efficacy), as well as IOP and endophthalmitis (as measurements of safety). No changes to trial outcomes after the trial commenced were performed. In the 2 weeks before surgery, all patients had a complete ophthalmologic examination including subjective refraction and corrected distance visual acuity (CDVA) measured using Snellen acuity charts, if the patient was unable to read any letter on the Snellen chart, hand motion or finger counting at a given distance were adopted. Results were converted to the logarithm of the minimum angle of resolution (logMAR) equivalent. Dilated fundus evaluation, Goldmann applanation tonometry, and OCT (Spectralis, Heidelberg Engineering GmbH) were performed, too. Patient characteristics, including age, sex, hemoglobin A1c (HbA1c) level, and diabetes control (diet and lifestyle modification- oral hypoglycemics- insulin), were recorded at baseline. The classification of diabetic retinopathy was in accordance with the International Clinical Diabetic Retinopathy Disease Severity Scale [27]. Central subfield macular thickness that corresponds to the mean macular thickness CSMT in the central 1.0 mm area was reported according to the ETDRS retinal thickness map [28].

Postoperative examinations, including CDVA and IOP measurements, were performed at 1 week and then at 1, 3, and 6 months. Dilated fundus and OCT examinations were performed at 1, 3, and 6 months. The same trained technician performed all OCT evaluations.

The study’s primary outcome was the difference between treatment groups with respect to central subfield macular thickness (CSMT) and diabetic retinopathy progression at the defined study points. Secondary outcomes included the difference between treatment groups regarding CDVA, IOP, endophthalmitis and the need for further interventions postoperatively.

### Escape treatment

Patients’ data were preserved in the department’s main computer as a file that is protected by a password and as a hard copy kept with the principal investigator for potential future intervention(s). In the case of clinically significant macular edema, patients were treated with an intravitreal injection with 1.25 mg (0.05 mL) bevacizumab, as reported by Flaxel et al. (OCT was used to quantitate centre or non-centre involving macular edema (CI-DME/ NCI-DME), clinical threshold for CI-DME was a central macular thickness 2 standard deviations above the normative study population of diabetics without macular edema) [29], or a visual acuity of less than 6/9.5 that is normally considered the threshold for injection in our hospital. From 12 weeks postoperatively, intravitreal or subtenon triamcinolone acetonide oranother bevacizumab injection was considered on a case-by-case basis in accordance with the treatment protocol in our hospital. Severe NPDR and any stage of PDR were treated with PRP laser as suggested by the DRS study [30]. Any IOP rise for more than 21 mmHg in 2 consecutive visits or more than 30 mmHg at any time point was considered elevated and was treated with anti-glaucoma eye drops, starting with a beta-blocker twice daily. If the rise persisted, the patient was excluded and transformed to the glaucoma clinic in our department.

Patients who developed severe postoperative inflammation, defined as at least 2C cells according to the SUN classification [31], received topical prednisolone acetate 1% q.2.h for 2 weeks. If severe inflammation persisted, dexamethasone phosphate 0.1% ± bromfenac 0.09% twice daily were added and the patient was transformed to the uveitis clinic in our department.

### Statistical analysis

Statistical analysis was performed using SPSS 25.0 (SPSS Inc., Chicago, IL, USA). Mean ± standard deviation (SD) was used for continuous variables and counts with percentages for categorical variables to describe patients’ characteristics at baseline. Kolmogorov- Smirnov test was used to check the normality of data distribution. For longitudinal comparisons of CSMT and its subgroups, IOP, and CDVA between baseline and each time point, the Friedman’s ANOVA test by ranks and ANOVA test for repeated measures were implemented with the Bonferroni correction and post-hoc analysis when needed. The chi-square and Fisher’s exact tests were used for categorical and nominal data. Linear regression models accounted for the correlation between repeated measurements within a patient and also include data from patients who dropped out of the study. Intention-to-treat analysis was adopted. A *p*-value < 0.05 was considered statistically significant, apart from cases where the Bonferroni correction was adopted. G power program v 3.1.3 was used to calculate the sample size. Alpha value was considered 0.05, statistical power of 85%, *P*-value was significant if ≤0.05. Comparison with local and international studies was conducted, too.

## Results

**Fig. 1:**
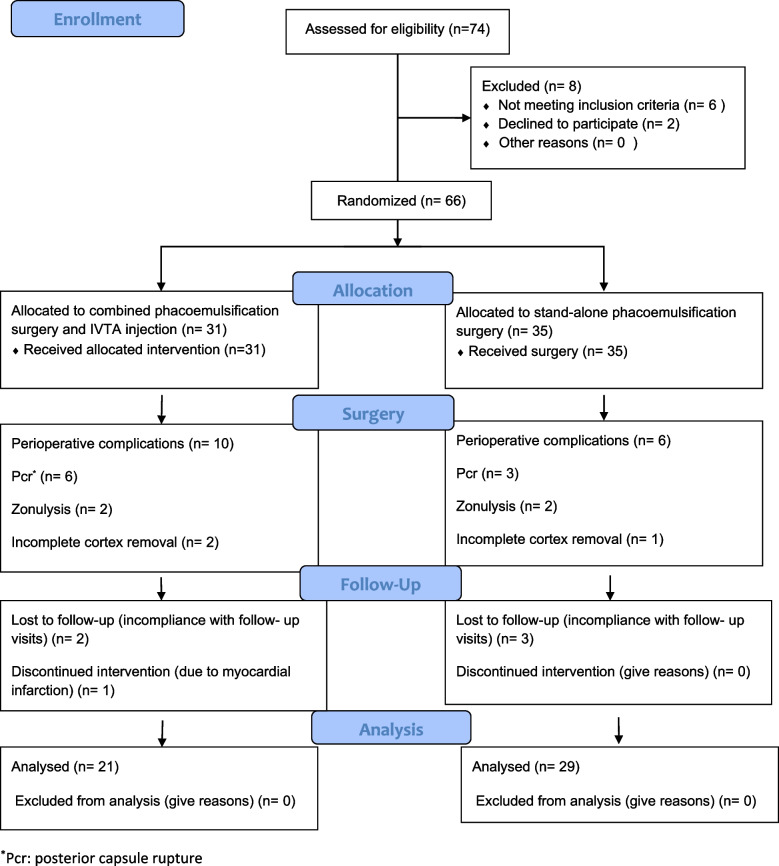
CONSORT flow diagram showing the number of participants who were screened, randomized and analyzed in the study. *Pcr: posterior capsule rupture

A total of 74 patients were assessed for eligibility as shown in the 2010 CONSORT flow diagram in Fig. 1, of whom; 66 patients were randomized (31 patients to the intravitreal triamcinolone injection IVTA group, and 35 patients to the no injection group). Data of 10 patients in the IVTA group and 6 patients in the no injection group, respectively were excluded from the final analyses because of perioperative complications as follows: posterior capsule rupture (*n* = 6 and 3), zonulolysis (*n* = 2 and 2), and incomplete cortex removal (*n* = 2 and 1). A total of 50 patients (21 in the IVTA group, and 29 in the no injection group) were included in the final analysis. Table 1 shows their baseline demographic and clinical characteristics. Of note is that the two groups were homogenous in terms of sex and age distribution (*p* > 0.05), as well as preoperative glycated haemoglobin percentage (*p* = 0.448) which reflects the uncontrolled glycemic status in both groups. Diabetes control method was classified into three categories:

1. Diet and lifestyle modification: a healthy diet that is low in refined sugar and fat in addition to physical activity of at least 30 minutes/3 days a week and smoking cessation (4.80, 10.30% in the IVTA and control groups, respectively without a statistically significant difference between them).
2. Oral hypoglycemic drugs (no restriction on the type) was the most frequent method of control (85.70, 82.80%, in the IVTA and control groups, respectively).
3. Insulin was the least used method (9.50, 6.90%, in the IVTA and control groups, respectively).

**Table 1:** Preoperative clinical and demographic data of study participants

			Case	control	*P* value
Sex^a^	male		47.60%	58.60%	0.567
	female		52.40%	41.40%	
Age (years)^b^	Mean ± SD		63.52 ± 12.38	63.69 ± 9.677	0.958
HbA1c%^b^	Mean ± SD		8.33 ± 1.83	8.71 ± 1.73	0.448
DM control^a^ method	diet and lifestyle		4.80%	10.30%	0.864
	oral medications		85.70%	82.80%	
	Insulin		9.50%	6.90%	
Cataract type & density	NS^c^	0	3.8%	2.3%	0.738
		+ 1	6.0%	5.0%	
		+ 2	75.7%	75.0%	
		+ 3	14.5%	17.7%	
	CS^d^	0	4.8%	6.7%	0.869
		+ 1	30.0%	30.0%	
		+ 2	39.5%	40.4%	
		+ 3	25.7%	22.9%	
	PSC^e^	0	9.5%	4.4%	0.884
		+ 1	20.3%	20.1%	
		+ 2	47.8%	50.2%	
		+ 3	22.4%	25.3%	

^a^Chi square test

^b^Student’s t test

^c^Nuclear sclerosis

^d^Cortical spikes

^e^Posterior subcapsular cataract

Cataract type and density were classified according to the WHO grading system [32]. NS + 2, CS + 2, PSC + 2 were the most frequent in both groups, without statistically significant difference between them (*p* > 0.05).

Three patients were lost to follow-up in the IVTA group (2 were incompliant with follow-up and 1 had a myocardial infarction) and 3 in the no injection group due to incompliance. Loss to follow-up equals 14.2 and 10.3% in the IVTA and no injection groups, respectively. Drop out was less than 15%, thus we imputed data using last observation carried forward (LOCF) and next observation carried backward (NOCB) method.

### Central subfield macular thickness (CSMT)

**Fig. 2:**
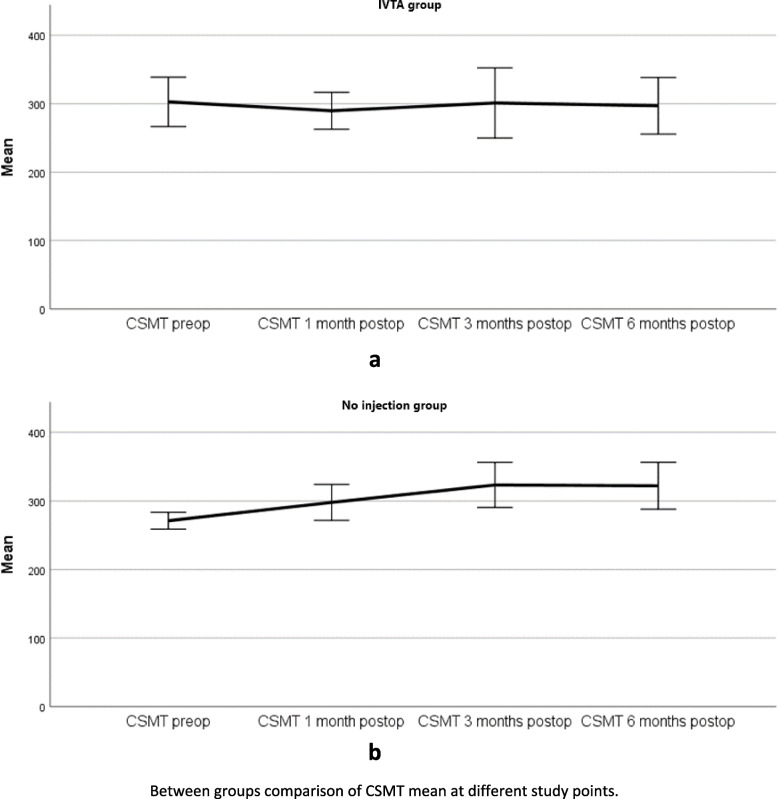
Simple line chart of CSMT mean comparison between groups at different study points. IVTA group (**a**), No injection group (**b**)

**Fig. 3:**
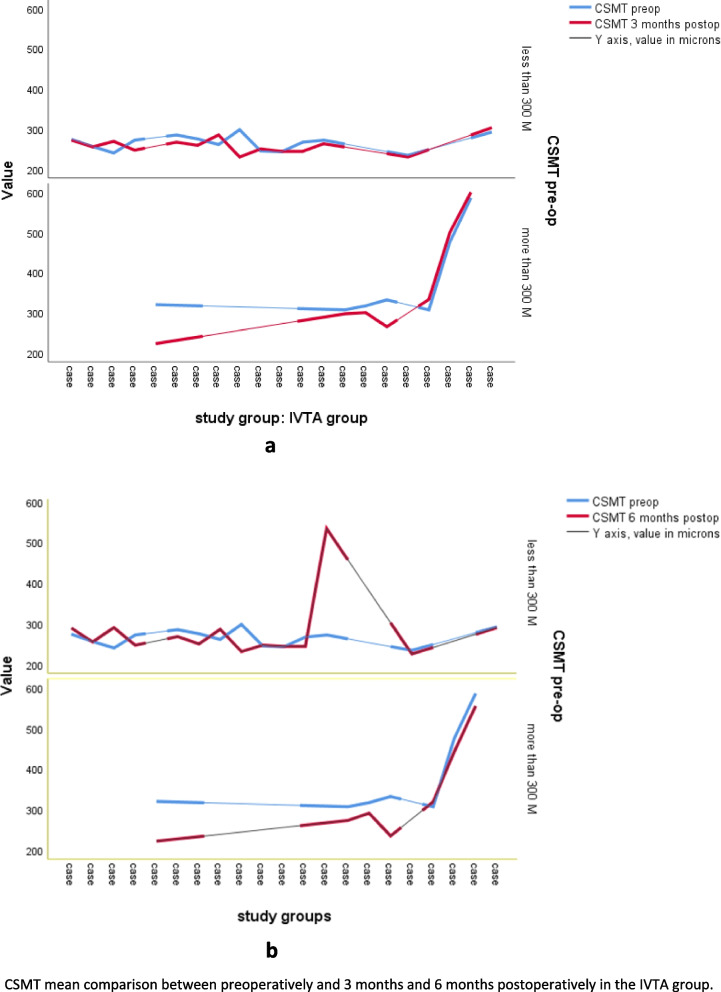
CSMT mean comparison between preoperatively and 3 months (**a**), and 6 months (**b**) postoperatively in the IVTA group

Table 2 shows the CSMT comparison between study groups at determined study points. Friedman’s ANOVA test by ranks was insignificant in the IVTA group (*p*- value = 0.342), and significant in the no injection group (*p* < 0.001). A postoperative CSMT increase of 30% from baseline in the no injection group in about 24% of patients was noted, while the values were distributed vaguely in the IVTA group as reflected in Fig. 2. Independent samples T-test, which was used to compare the change in CSMT between study groups at determined study points revealed no significant statistical difference in thicknesses at any study point (*p* > 0.05 at all study points). A repeated-measures ANOVA with a Greenhouse-Geisser correction after linearity checking determined that the mean CSMT value differed statistically significantly between time points in the IVTA group for a value (F (2.281, 111.761) = 347.300, *P* < .0001). This could be translated into a significant change in CSMT postoperatively when preoperative CSMT was no less than 347.3 μm. Post hoc analysis with a Bonferroni adjustment revealed that CSMT value was statistically significantly different between pre-op and 3 months post-op (269.340 μm; 95% CI, 245.768- 292.912), 1 month post-op and 3 months post-op (279.400; 95% CI, 253.416- 305.384), as well as between 3 months post-op and 6 months post-op (296.540; 95% CI,260.474- 332.606) with a *p*-value< 0.0001 for each. Linear regression models showed a moderate correlation between IVTA injection and CSMT value with a 25.8% decrease (*p* < 0.0001) and a 26% decrease (*p* = 0.0001) in CSMT based on R^2^ value at 3- and 6-months post-op, respectively. At 1-month post-op, this correlation was fair (17% reduction in CSMT, *p* = 0.01). Further subgroups analysis was carried out using Friedman’s ANOVA test by ranks. The result was statistically significant for CSMT values of 300 μm (*p* = 0.04) as shown in Fig. 3, and linear regression analysis confirmed a very strong correlation (R = 0.995, R2 = 0.913). This result is compatible with the repeated measures ANOVA test which designated 347.300 μm as significant. Consequently, we can consider CSMT = 347.300 μm a cut-off value in the IVTA group.

**Table 2:** Between groups comparison of CSMT^a^ at different study points

		CSMT pre-op	CSMT 1 month post-op	CSMT 3 months post-op	CSMT 6 months post-op
IVTA group	Mean ± Std. Deviation	302.81 ± 82.570	289.71 ± 61.957	301.14 ± 117.546	297.05 ± 94.834
	Mean rank^b^	2.86	2.57	2.40	2.17
	*P*-value (overall)	0.342			
No injection group	Mean ± Std. Deviation	271.21 ± 33.155	298.03 ± 70.587	323.48 ± 88.413	322.28 ± 91.956
	Mean rank	1.66	2.38	2.84	3.12
	*P*-value (overall)	0.000			

^a^*CSMT* Central subfield macular thickness

^b^Friedman’s ANOVA test by ranks

*P*-value is significant at the level *p* < 0.001

### Diabetic retinopathy (DR) progression

**Fig. 4:**
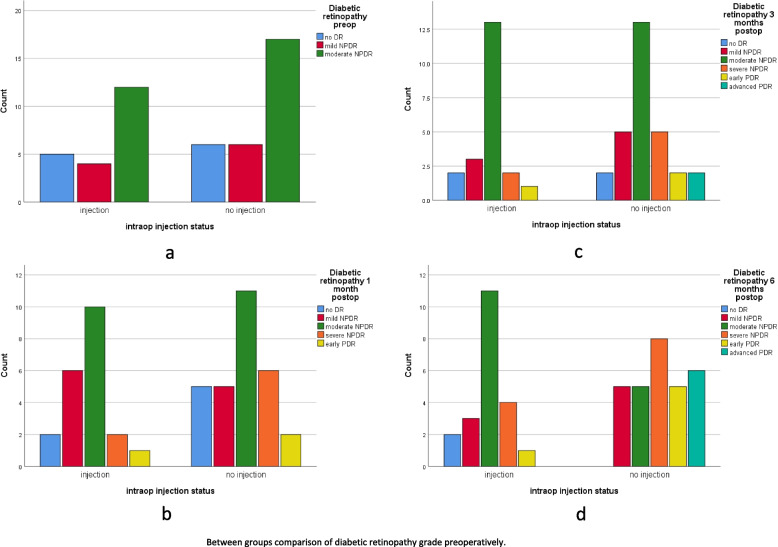
Bar charts comparing the grade of diabetic retinopathy between groups. Preoperatively (**a**), 1-month postoperatively (**b**), 3-month postoperatively (**c**), 6-month postoperatively (the figure here is dropped out) (**d**)

Table 3 shows the percentage of each degree of diabetic retinopathy DR within the study group and within diabetic retinopathy at the determined study points. We used Fisher’s exact test to evaluate the relationship between study groups and DR progression. No statistically significant association was obtained at any study point (*p*-value> 0.05) except at 6 months post-op (*p* = 0.012). Cramer’s V test was applied to evaluate the strength of this association at 6 months which seemed to be moderately strong (Cramer’s V = 0.526). Moderate NPDR was most prevalent in the IVTA group at 6 months compared to severe NPDR and early PDR in the no injection group. Figure 4 shows the distribution of DR grades at different study points.

**Table 3:** Between groups comparison of diabetic retinopathy progression at different study points

			No DR	Mild NPDR	Moderate NPDR	Severe NPDR	Early PRD	High-risk PDR	*P* value (overall)
Diabetic retinopathy pre-op	IVTA group	Count	5	4	12	0	0	0	1.00
		% within group	23.8%	19.0%	57.1%	0%	0%	0%	
		% within diabetic retinopathy	45.5%	40.0%	41.4%	0%	0%	0%	
	No injection group	Count	6	6	17	0	0	0	
		% within group	20.7%	20.7%	58.6%	0%	0%	0%	
		% within Diabetic retinopathy preop	54.5%	60.0%	58.6%	0%	0%	0%	
Diabetic retinopathy 1-month post-op	IVTA group	Count	2	6	10	2	1	0	0.713
		% within group	9.5%	28.6%	47.6%	9.5%	4.8%	0%	
		% within Diabetic retinopathy	28.6%	54.5%	47.6%	25.0%	33.3%	0%	
	No injection group	Count	5	5	11	6	2	0	
		% within group	17.2%	17.2%	37.9%	20.7%	6.9%	0%	
		% within Diabetic retinopathy	71.4%	45.5%	52.4%	75.0%	66.7%	0%	
Diabetic retinopathy 3 months post-op	IVTA group	Count	2	3	13	2	1	0	0.792
		% within group	9.5%	14.3%	61.9%	9.5%	4.8%	0%	
		% within Diabetic retinopathy	50.0%	37.5%	50.0%	28.6%	33.3%	0%	
	No injection group	Count	2	5	13	5	2	2	
		% within group	6.9%	17.2%	44.8%	17.2%	6.9%	6.9%	
		% within Diabetic retinopathy	50.0%	62.5%	50.0%	71.4%	66.7%	100%	
Diabetic retinopathy 6 months post-op	IVTA group	Count	2	3	11	4	1	0	0.012
		% within group	9.5%	14.3%	52.4%	19.0%	4.8%	0.0%	
		% within Diabetic retinopathy	100.0%	37.5%	68.8%	33.3%	16.7%	0.0%	
	No injection group	Count	0	5	5	8	5	6	
		% within group	0.0%	17.2%	17.2%	27.6%	17.2%	20.7%	
		% within Diabetic retinopathy	0.0%	62.5%	31.3%	66.7%	83.3%	100%

### Corrected distant visual acuity (CDVA)

Table 4 shows a CDVA comparison between groups at different study points. We can easily see that CDVA was significantly improved from baseline 6/60; (Logarithm of the Minimum Angle of Resolution logMAR 1.0) pre-op to 6/6 (logMAR 0.00) at 6 months postop in the IVTA group and from baseline 6/120 (logMAR 1.3) pre-op to 6/12 (logMAR 0.3) at 6 months postop in the control group. The gain in visual acuity was significantly higher in the IVTA group at 6 months postoperatively (*p* < 0.001). This association is reflected in the ascending values of Friedman’s ANOVA test ranks that are elucidated in Table 4. We can speculate how the IVTA group ranks are always higher than the no injection group, especially at 6 months post-op (4.60 vs 3.78). The linear regression model was statistically significant at 6 months post-op and showed a moderate correlation with about 27.3% gain in CDVA in the IVTA group.

**Table 4:** Between groups comparison of CDVA^a^ at different study points

		CDVA pre-op	CDVA 1 week post-op	CDVA 1 month post-op	CDVA 3 months post-op	CDVA 6 months post-op
IVTA group	Mean logMar (Snellen)	1.0 (6\60)	0.5 (6\19)	0.4 (6\ 15)	0.3 (6\ 12)	0.0 (6\6)
	Median logMAR (snellen)	1.0 (6/60)	0.5 (6\19)	0.4 (6/15)	0.2 (6/9.5)	-0.1 (6/4.8)
Mean rank^b^		1.36	2.24	3.21	3.60	4.60
No injection group	Mean logMAR (Snellen)	1.3 (6\120)	0.7 (6\30)	0.5 (6\19)	0.4 (6\15)	0.3 (6\12)
	Median logMAR (snellen)	1.3 (6\120)	0.7 (6/30)	0.4 (6/15)	0.4 (6\15)	0.4 (6/15)
Mean rank		1.78	2.76	3.29	3.40	3.78
*P* value (overall)		0.38	0.56	0.20	0.06	0.000^c^

^a^*CDVA* Corrected distant visual acuity

^b^Friedman’s ANOVA test by ranks

^c^*P*-value is significant at the level *p* < 0.001

**Fig. 5:**
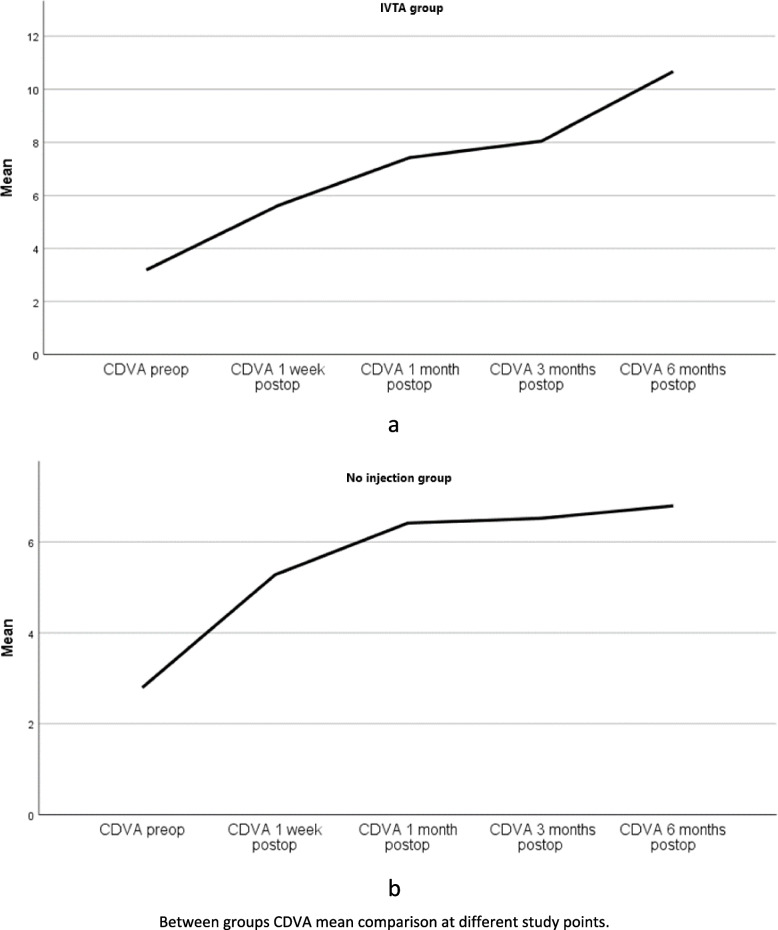
Simple line chart of CDVA mean comparison between groups at different study points. IVTA group (**a**), No injection group (**b**)

Notably, a strong relationship (*p* < 0.001) exists between CDVA and both CSMT and diabetic retinopathy progression at all the determined study points. Figure 5 shows the distribution of CDVA means at different study points.

### Intraocular pressure (IOP)

**Fig. 6:**
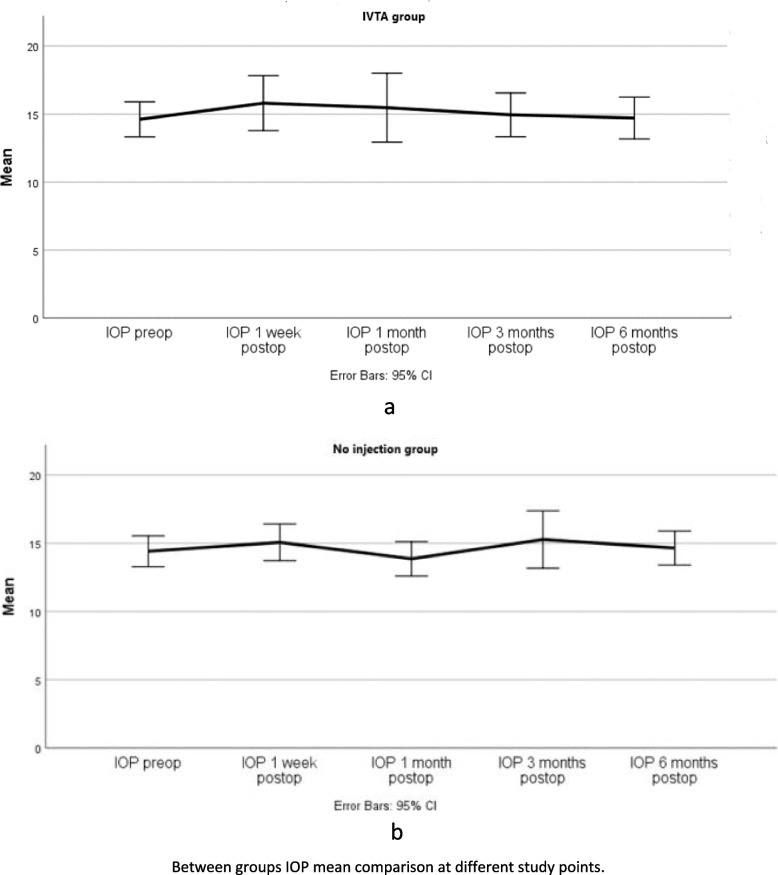
Simple line chart of IOP mean comparison between groups at different study points. IVTA group (**a**), No injection group (**b**)

Table 5 shows the IOP comparison between groups. The repeated measures ANOVA test was statistically insignificant at all the determined study points (F (4, 192) = 1.417, *P* = 0.230). Sphericity was checked before carrying on the ANOVA to study the within elements effect (Mauchly: *P* = 0.4). Therefore, no momentous IOP elevation existed in our study. Although 5 patients in each study group were taking anti-glaucoma eye drops, 3 had their IOP controlled with eye drops before surgery. Only 2 in each group started the eye drop after surgery at different study points. None had an IOP > 30 mmHg, and we could manage the IOP rise with medications successfully in all the aforementioned patients. Figure 6 shows the distribution of IOP means at different study points.

**Table 5:** Between groups comparison of IOP^a^ at different study points

	Mean ± Std. Deviation				
	IOP pre-op	IOP 1 week post-op	IOP 1 month post-op	IOP 3 months post-op	IOP 6 months post-op
IVTA group	14.62 ± 2.94	15.81 ± 4.63	± 15.485.81	14.95 ± 3.68	14.71 ± 3.52
No injection group	14.41 ± 2.96	15.07 ± 3.6	13.86 ± 3.409	15.28 ± 5.66	14.66 ± 3.35
*P*-value	0.812	0.529	0.223	0.820	0.952

^a^*IOP* Intraocular pressure

### The need for further intervention(s)

#### Eye drops

Table 6 shows the categories of eye drops studied (anti-glaucoma, NSAIDs, corticosteroids, combined eyedrops) and their distribution between study groups. There was no significant relationship between their usage and the study groups (Fisher’s exact test, *P*-value = 0.58).

**Table 6:** Between groups comparison of the need for further eyedrop(s) post-surgery

			Frequency	Percent%
Eye drops	IVTA group	None	15	71.4%
		Antiglaucoma	5	23.8%
		NSAIDs^a^	1	4.8%
	No injection group	None	19	65.5%
		Antiglaucoma	3	10.3%
		NSAIDs	4	13.8%
		Corticosteroids	1	3.4%
		NSAIDs+ Antiglaucoma	1	3.4%
		corticosteroids+ Antiglaucoma	1	3.4%
*P* value			0.58	

^a^Non-steroidal anti-inflammatory

### Pan-retinal photocoagulation (PRP) and further injection(s)

Table 7 compares the need for PRP and injections between study groups. We can see that more patients in the no injection group required PRP laser post-surgery (27.6% vs 9.5%, respectively). Despite this, no statistically significant relationship was obtained between the procedure and the study groups (Fisher’s exact test, *P*-value = 0.16).

**Table 7:** Between groups comparison of the need for further intervention(s) post-surgery

			Frequency	Percent
Post-op injection(s)	IVTA group	none	16	76.2%
		IVB^a^	3	14.3%
		IVB+ IVTA	2	9.5%
	No injection group	none	15	51.7%
		IVB	12	41.4%
		IVTA	1	3.4%
		STTA^b^	1	3.4%
*P* value			0.04	
PRP^c^	IVTA group	none	19	90.5%
		PRP	6	9.5%
		none	21	72.4%
	No injection group	PRP	8	27.6%
*P* value			0.16	

^a^Intravitreal bevacizumab

^b^Subtenon triamcinolone acetonide

^c^Pan-retinal photocoagulation

On the contrary, the different types of injection (intravitreal bevacizumab IVB, intravitreal triamcinolone acetonide IVTA, subtenon triamcinolone acetonide STTA) were statistically significantly more required in the no injection compared to the IVTA group (48.3% vs 23.8%, respectively), (Fisher’s exact test, *P*-value = 0.04).

#### Endophthalmitis

No incidence of endophthalmitis was recorded during the 6-month follow-up in our study.

## Discussion

Phacoemulsification surgery in diabetic patients has been a field of controversy for a long time. While some studies suggest that uneventful phacoemulsification surgery seems to be innocent of provoking diabetic retinopathy (DR) progression or diabetic macular edema (DME) exacerbation outside the natural course of the disease [33], others claim that cataract surgery may accelerate the progression of pre-existing DR, induce rubeosis, precipitate or initiate DME [5].

Our results showed a trend towards increased central subfield macular thickness CSMT of 30% from baseline CSMT in the no injection group after the surgery. In the intravitreal triamcinolone injection IVTA group, we noticed a decrease in CSMT on OCT that was most significant at 3- and 6-months follow-up visits and was most noteworthy when pre-op CSMT value was ≥300 μm with a cut-off value of baseline CSMT at 347.3 μm where the reduction was utmost. One can notice the trend toward relatively high values of thickness even preoperatively in our patients. Kwon et al. reported that after cataract surgery 18% of patients with diabetic retinopathy developed thickening of more than 30% of the CSMT, which correlated to the severity of retinopathy [34]. Kim et al. reported thickening for more than 30% in 22% of the participants (11 out of 50 patients had their macular thickness increased from 171 μm preoperatively to 373 μm at 1 month, experienced a significant loss of vision, and demonstrated cystoid abnormalities. Interestingly, preoperative, intraoperative, and postoperative medical management was left to the individual decision-making of the surgeon) [13]. Krepler et al. reported that 31% of eyes with NPDR developed CSMO after cataract surgery through 1 year of follow-up (Postoperative treatment consisted of betamethasone, neomycin (BetnesolN) and diclofenac eyedrops (Voltaren Ophtha) four times a day and BetnesolN ointment at night for 4 weeks) [35]. A 30-μm thinner CSMT and better visual acuity in the initial-IVTA group than in the initial placebo group were reported by Gilles et al. (who retreated eyes with a reduction of visual acuity of at least 5 letters from previous peak value and persistent CMT greater than 250 μm with IVTA, focal or grid macular laser treatment was administered from the third year on) [36]. The percentage of patients with overall CME in our study raises to about 36% should we adopt the ESCRS PREMED study definition for CME; that is, at least a 10% increase in the mean central subfield macular thickness compared with the preoperative baseline on OCT [37]. While we found a CSMT value of 300 μm to be a threshold for benefiting from treatment, the UK Diabetic Retinopathy Electronic Medical Record Users Group defined a CMT > 400 μm as vulnerable to developing a treatment requiring CME one-year post-op [38]. Intravitreal triamcinolone acetonide has been claimed to be a potent steroid in reducing CSMT and CME by many researchers [39–41]. There is conflicting evidence on a wide variety of treatments and various means of delivery that have been investigated regarding pseudophakic macular edema treatment and prophylaxis; such as subtenon TA [25], intravitreal anti- VEGF [42, 43], corticosteroid implants [44], and topical NSAIDs alone or combined with topical steroids eye drops [45]. Nepafenac 0.3%, in particular, may exhibit activity against DME and improve visual acuity outcomes [46] Recently, intracameral dexamethasone has been investigated with claimed short-term efficacy in CMT reduction in diabetic patients [47]. None has shown superiority and the effect seems to correlate strongly with the pre-op macular status and the variation in the drive between diabetic patients with and without ocular manifestations [48, 49]. Of notice, triamcinolone acetonide has shown some superiority in longevity -compared to anti-VEGF- in the DIMECAT trial, EURETINA guidelines, and compared to placebo in Gilles et al. 5-year follow-up trial [36, 43, 50].

Due to similarities in methodology and results with this study, it is worth mentioning the ESCRS PREMED study separately; a European multi-centre RCT. In the first report [45], non-diabetic patients who had uneventful phaco surgery were randomized into three subgroups (topical bromfenac 0.09%, topical dexamethasone 0.1%, or both), after 12 weeks of follow-up, they concluded that the combination arm of the study had the lowest incidence of CME compared to dexamethasone alone with the highest incidence. In our study, however, corticosteroid eye drop was used solely as a matter of guideline in the hospital, in addition to the high cost of NSAIDs eye drops. In the second report [25], diabetic patients were randomized to receive no injection or a subconjunctival TA 40 mg or 1.25 mg of IVB injection, or both injections at the end of phacoemulsification. They found that patients who received subconjunctival TA did not develop CME at any time point during the 12-week follow-up, in addition to being privileged with lower macular thickness and volume at 6 and 12 weeks postoperatively, but underprivileged with a significant increase in IOP that was not observed in the other subgroups. The high IOP could be alleviated medically, except for one patient who required surgical intervention to remove the TA depot. On the contrary, IVB had no significant effect on macular thickness or volume. Our study results support the assumption of TA efficacy in a different route of administration. Moreover, although higher IOP was observed in the IVTA group, the increase was insignificant and could be controlled medically. This study had a longer follow-up period of 6 months, as well.

Evidence suggests that a sharp increase in treatment-requiring DME after cataract surgery for all grades of DR, peaks in the 3 to 6 months postoperative period [30]. However, the standard of care is still lacking [38].

Our data suggest that IVTA at the end of surgery could slow down the progression of diabetic retinopathy. This effect is most prominent at 6 months postoperatively. Most of the patients in the intervention group fall within the moderate NPDR category at 6-month follow-up, while severe NPDR followed by early PDR was more prevalent in the control group. In addition, fewer patients in the IVTA group required PRP laser and further intravitreal injection(s) during the follow-up period, which goes in line with the efficacy of IVTA. This effect has been well documented in the DRCR.net study that demonstrated slower progression from NPDR to PDR in the IVTA compared with macular laser treatment [51], and the Pan American Collaborative Retina Study Group [41]. Improved DR grading with dexamethasone implants has been documented by the OZDRY study [52]. BEVORDEX trial showed a relatively low rate of new PDR events over 2 years in eyes that were treated with either intravitreal dexamethasone implant or bevacizumab [53]. Blunted DR progression at 6 months postoperatively has also been reported by Cheema et al. with intravitreal bevacizumab injection during cataract surgery [54]. Nonetheless, some authors contradict the triamcinolone enhancing effect on DR progression when delivered either intravitreally [40] or subtenon [55].

We found that visual acuity improvement was significantly related to the CSMT and DR progression in all phases of follow-up with maximal gains arising 6 months post-op. Although CDVA was enhanced in both study groups, a greater upshot was observed in the IVTA group compared to the control group. There seems to be harmony between different studies that visual acuity results after phacoemulsification are generally good in diabetic patients despite being somehow suboptimal [20]. The European Registry of Quality Outcomes for Cataract and Refractive Surgery analysis revealed that 28% of eyes with DR had worse VA after cataract surgery compared to 11.9% of those without ocular co-morbidities [19]. Surgical inexperience may be a determinant factor of poor visual results side-by-side with retinopathy status before surgery [18]. Intravitreal TA [56, 57], or bevacizumab [41, 56] injections were suggested to improve visual outcomes in diabetic patients. However, this effect may be exclusive to when no pre-op DR exists [58], or may wean after 6 months of surgery as suggested by Ahmadabadi et al. who did not find a statistically significant difference in VA despite a tendency to be better in the treatment group [40].

No substantial intraocular pressure swings were noted in our patients. Even after IVTA injection, a transit insignificant rise of no more than 30 mmHg was recorded. There were no statistically significant differences between the 2 groups at any postoperative visit. No patients needed glaucoma surgery and all cases complied with medical therapy. Our results go in accord with Campos et al. who used 3.2 mg of IVTA instead of 4 mg [57], and Ahmadabadi et al. who suggests that a single injection of IVTA would not raise IOP to a harmful level should it provoke a rise [40], and Habib et al. who encountered IOP rise of up to 34 mmHg but all were controlled with eye drops [59]. On the contrary, a significant IOP rise in 23.5% of the participants has been documented by Lam et al. [60].

Furthermore, our study recorded no incidence of endophthalmitis during the 6 months follow-up. We might suggest that this is the norm for uneventful surgery based on our results and other researchers’ results [40, 57, 59]. Endophthalmitis and noninfectious endophthalmitis are claimed to be complications of all intravitreal injections and not of IVTA itself [26]. Besides, cataract surgery can be an ideal setting for combining the two procedures in order to reduce the patient’s potential risk of endophthalmitis from two separate intraocular episodes to one that is performed under surgically sterile circumstances with full control of the globe and intraocular pressure, which may offer improved patient convenience [40, 59]. In addition, combining phacoemulsification and TA has shown clear cost-effectiveness in the ESCRS PREMED study report 6 [61]. This is of significant impact in places with limited resources and low-income countries where more costly alternatives availability is restricted.

Limitations to this study include the relatively small sample size and the uncontrolled preoperative glycemic status in most study participants. In addition, we only studied the central 1.0 mm zone of the ETDRS map which was relatively thick at baseline in both groups. Furthermore, postoperative treatment(s) were based on a case-by-case basis by the un-blinded investigator.

## Conclusion

In conclusion, our results suggest that phacoemulsification surgery is efficient and feasible in diabetic patients with variable degrees of diabetic retinopathy. Triamcinolone acetonide is a relatively cost-effective and affordable substance with minimal side effects. This point is paramount in low-to-middle income countries where more expensive alternatives to triamcinolone may not be obtainable. This study may provide evidence to support the usage of intravitreal triamcinolone acetonide combined with phacoemulsification to optimize the results regarding visual acuity and central macular thickness and slow down diabetic retinopathy progression afterwards. Further prospective randomized studies are warranted to establish a crystal-clear definition of OCT diabetic macular edema and to extensively analyze the interaction between each stage of diabetic ophthalmopathy and phacoemulsification surgery. In addition, measuring the effect of postoperative topical steroids and NSAIDs, and pre-operative or concurrent anti-VEGF injections is required to extract the true value of IVTA at the time of surgery. Consequently, “phaco enhancers” could be utilized wisely.

## Data Availability

The dataset supporting the conclusions of this article is owned by Damascus University, Damascus, Syria. A copy of the data can be requested by email. Email: info@damascusuniversity.edu.sy.

## Abbreviations

CSMT: Central subfield macular thickness
DR: Diabetic retinopathy
CDVA: Corrected distant visual acuity
IOP: Intraocular pressure
IVTA: Intravitreal triamcinolone acetonide injection
STTA: Subtenon triamcinolone acetonide injection
IVB: Intravitreal bevacizumab
PRP: Pan-retinal photocoagulation
